# iPSC model of human specific neuroinflammation in Alzheimer’s disease

**DOI:** 10.1002/alz70861_108756

**Published:** 2025-12-23

**Authors:** Ivanna Ihnatovych, Ryu Platinum Dorn, Robin Schwartz, Ruea‐Yea Huang, Kinga Szigeti

**Affiliations:** ^1^ University at Buffalo, Buffalo, NY USA

## Abstract

**Background:**

Alzheimer’s disease (AD) is a human specific neurodegenerative disorder characterized by loss of memory and cognitive functions associated with amyloid beta 1‐42 (Aβ_1‐42_) plaques and tau fibrils. In the brain, an elevated level of Aβ_1‐42_ leads to neuronal death, microglia activation and neuroinflammation. The α7 nicotinic acetylcholine receptor (α7nAChR) binds Aβ_1‐42_ with high affinity. *CHRFAM7A*, a human restricted fusion gene, incorporates into the α7nAChR pentamer creating a hypomorphic α7/CHRFAM7A nAChR. We examine how the humanized receptor affects Aβ_1‐42_ induced neuroinflammation.

**Method:**

To study the effect of Aβ_1‐42_ on neuronal and immune cells two models were utilized: human isogenic iPSC model that includes medial ganglionic eminence (MGE) progenitors and microglia like cells (MGL) differentiated from *CHRFAM7A* null, *CHRFAM7A* nascent and isogenic *CHRFAM7A_*KI cell lines; and human AD and age matched control macrophages (28 donors).

**Result:**

Consistent with a hypomorphic receptor, Aβ_1‐42_ uptake was mitigated in a dose‐dependent manner in *CHRFAM7A* carriers in both MGE progenitors and MGL as demonstrated by flow cytometry, ELISA, and immunofluorescence. Interestingly, a decreased Aβ_1‐42_ uptake resulted in significantly elevated innate immune cytokine expression (interleukin 1beta, interleukin 6, and tumor necrosis factor alpha) in *CHRFAM7A* carriers compared to null. This finding was consistent with release of the α7nAChR‐mediated anti‐inflammatory effect. Additionally, higher NF‐κB activation in *CHRFAM7A* carriers compared to null was observed in MGL as shown by a prolonged p65 translocation to the nucleus (immunofluorescence) and an increased p65 phosphorylation (immunoblot). Human variance of Aβ_1‐42_ effect was characterized in primary human macrophages. Similar to the iPSC model, Aβ_1_‐42 treatment led to an increased cytokine level in *CHRFAM7A* carriers compared to non‐carriers.

**Conclusion:**

We found dual effect of *CHRFAM7A* on Aβ_1‐42_ associated pathology in AD: it provides protection against toxic concentration of Aβ_1‐42_ by decreased uptake through the humanized α7/CHRFAM7A nAChR and increases the innate immune response by switching the α7nAChR from an anti‐inflammatory to pro‐inflammatory transducer. Our iPSC model presents an opportunity to elucidate the molecular mechanism of these processes and establish high throughput screens.

